# Stereotactic Body Radiotherapy-Induced Arterial Hypervascularity of Non-Tumorous Hepatic Parenchyma in Patients with Hepatocellular Carcinoma: Potential Pitfalls in Tumor Response Evaluation on Multiphase Computed Tomography

**DOI:** 10.1371/journal.pone.0090327

**Published:** 2014-02-28

**Authors:** Mee Jin Park, So Yeon Kim, Sang Min Yoon, Jong Hoon Kim, Seong Ho Park, Seung Soo Lee, Yedaun Lee, Moon-Gyu Lee

**Affiliations:** 1 Department of Radiology and Research Institute of Radiology, University of Ulsan College of Medicine, Asan Medical Center, Seoul, Korea; 2 Department of Radiation Oncology, University of Ulsan College of Medicine, Asan Medical Center, Seoul, Korea; Yonsei University College of Medicine, Korea, Republic of

## Abstract

**Purpose:**

To evaluate temporal changes in contrast enhancement patterns of non-tumorous hepatic parenchyma with a focus on arterial hypervascularity on multiphase computed tomography (CT) in patients with hepatocellular carcinoma (HCC) treated with stereotactic body radiotherapy (SBRT).

**Methods:**

We retrospectively identified 61 patients who had undergone multiphase contrast-enhanced CT at one, three, and six months after SBRT. Irradiated versus non-irradiated liver parenchyma was delineated by cross-correlation with the dose-volume histogram of SBRT plan. Serial changes in the contrast enhancement patterns of the irradiated versus non-irradiated liver parenchyma were evaluated by two abdominal radiologists in consensus. We compared the frequency of the contrast enhancement patterns according to the follow-up period using the Fisher-Freeman-Halton exact test.

**Results:**

The irradiated non-tumorous hepatic parenchyma showed that the prevalence of arterial hypervascularity increased during the follow-up period (*P*<.01): 11.5% (7/61) in one, 45.9% (28/61) in three, and 54.1% (33/61) in six months. Contrast wash-out on the delayed phase was uncommon: 1.6% (1/61) in one, 3.3% (2/61) in three, and 0% in six months.

**Conclusion:**

The incidence of arterial hypervascularity of the irradiated hepatic parenchyma gradually increased until six months after SBRT, which could interfere with the accurate evaluation of treatment response. The lack of wash-out on the delayed phase in the hypervascular area would distinguish SBRT-related change from residual/recurred HCC.

## Introduction

Hepatocellular carcinoma (HCC) is a major health problem worldwide, with more than half a million new cases yearly [1]. Traditional treatment modalities for HCC include surgical resection, percutaneous ablation, and transarterial chemoembolization [2]. Stereotactic body radiotherapy (SBRT) is an emerging treatment option for patients with inoperable HCC because it offers a high local tumor control probability and a high safety profile [3]–[5]. SBRT is a technique that uses precisely targeted three-dimensional conformal radiation delivered via multiple portals in various directions in a hypofractionated regimen under image guidance [6]. Although SBRT is designed to accurately deliver a high dose of radiation to a confined target while minimizing radiation to normal tissue [3], [7], [8], neighboring non-tumorous hepatic parenchyma are exposed to irradiation and may thus present with radiation-induced changes.

Many previous imaging studies on radiation-induced changes of the liver reported changes associated with conventional radiotherapy rather than SBRT [9]–[14]. Radiation-induced changes associated with SBRT are expected to differ from those associated with conventional radiotherapy because of the complex and distinct mode of radiation dose delivery of SBRT as well as delivered radiation dose [15]. With the increasing use of SBRT, it is important to be familiar with the radiation-induced changes of the liver after SBRT on multiphase computed tomography [16], as this information will help us to accurately assess treatment responses [17]–[21].

There have been a few published studies regarding the multiphase CT findings of non-tumorous hepatic parenchyma after SBRT [16], [22]–[24]. Two of these studies briefly mentioned arterial hypervascularity as a form of focal liver reaction in the irradiated hepatic parenchyma, but without describing the incidence or temporal changes of arterial hypervascularity [16], [22]. As arterial hypervascularity is also a key diagnostic feature of HCC [25], we need to determine the characteristics of arterial hypervascularity of non-tumorous hepatic parenchyma after SBRT and differentiate them from recurred or residual HCC. Considering that multiple follow-up CT scans are generally available following the treatment of HCC, we suggest that temporal changes in the enhancement patterns of the irradiated hepatic parenchyma are also important to note.

Therefore, the purpose of our study is to evaluate temporal changes in contrast enhancement patterns of non-tumorous hepatic parenchyma on multiphase CT with a focus on arterial hypervascularity in patients with HCC treated with SBRT.

## Materials and Methods

The institutional review board of Asan Medical Center approved this study, and informed consent was waived due to the retrospective nature of the study. All the data of patient records and information were anonymized and de-identified prior to analysis.

### Study Patients

A computerized search of our institution's medical records revealed 93 patients (mean age ± SD, 60.8±7.3 years), including 75 men (60.3±7.0 years) and 18 women (62.9±8.4 years), with HCC treated using SBRT between March 2007 and December 2009. At our institution, indication of SBRT for the patients with HCC was as follows: (1) HCC not suitable for surgery or percutaneous ablative therapies; (2) HCC confined to the liver without extrahepatic metastases; HCC less than 6 cm in the longest diameter or up to 3 lesions; (4) HCC with no evidence of major vascular invasion; (5) a liver function of Child-Pugh class A or B; (6) adequate residual functional liver volume (more than 700 cc); and (7) a sufficient distance (more than 2 cm) from the adjacent organs at risk such as duodenum, stomach, colon, and spinal cord. The diagnosis of HCC was made non-pathologically according to the noninvasive diagnostic criteria proposed by the American Association for the Study of Liver Diseases in 93 patients [26].

Among these 93 patients, 65 had follow-up multiphase CT scans one, three, and six months after SBRT. Four of the 65 patients were excluded because of potentially confounding processes affecting contrast enhancement patterns of the irradiated hepatic parenchyma, i.e. (1) ethanol injection during the follow-up period (n = 2), (2) metal artifact from the gold fiducial markers detected in the liver and thus impairing the image quality in a very small target volume (n = 1), and (3) portal vein thrombus causing hepatic perfusion abnormality (n = 1). The remaining 61 patients (mean age ± SD, 61.7±8.3 years), including 52 men (61±8 years) and nine women (65.6±9.4 years), were finally included in the analyses.

In the study population, 50 of these had a single lesion and the remaining 11 patients had two or more lesions. For the 11 patients with multiple HCCs, only the largest lesion was included in further analysis to avoid clustering effects. The size of the 61 HCCs ranged from 0.3 to 3.7 cm (mean size ± SD, 1.8 cm±0.9 cm). Among the 61 HCCs, 19 HCCs were treated with transcatheter arterial chemoembolization (TACE) in combination with SBRT for the lesion of interest. Child-Pugh class of liver cirrhosis was also assessed one, three and six months after SBRT.

### Treatment with SBRT

**Figure 1:**
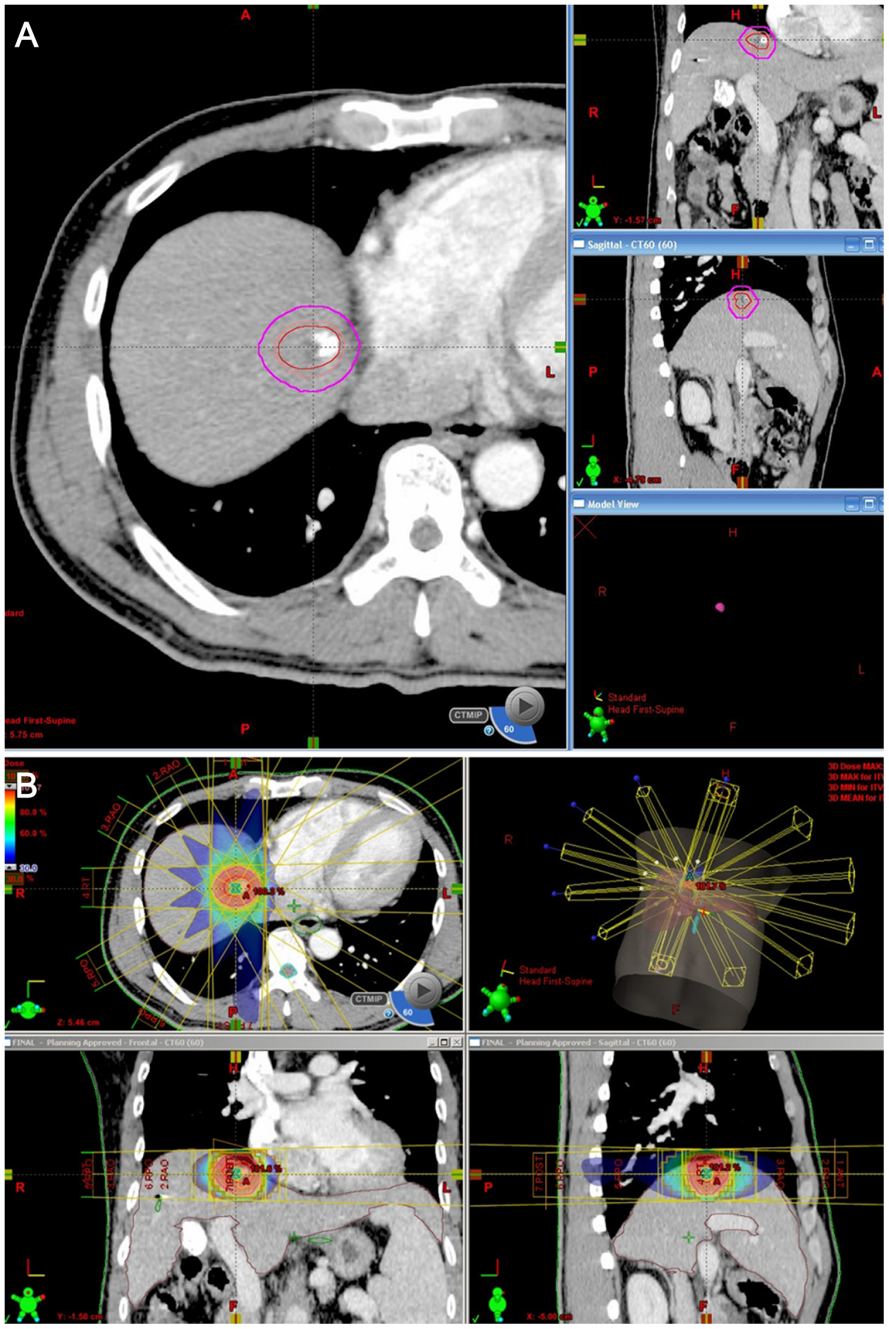
An example of delineation of the target volume and treatment planning: Planning for SBRT. (A) The planning target volume (purple circle) is expanded to cover the gross tumor volume (red circle). (B) Using six coplanar photon beams, a highly conformal isodose distribution is obtained three-dimensionally. The dose distribution is demonstrated on the color map as a percentage of the total radiation dose.

All patients underwent CT-based simulation (LightSpeed®RT 1, GE, Milwaukee, WI, USA) for SBRT. Prior to CT simulation, gold fiducial markers were inserted under US-guidance surrounding the target tumor. Implanted markers are typically separated by 3∼5 cm in distance between markers and they indicate the location of tumors for SBRT. CT simulation was performed with free breathing using a four-dimensional (4D) CT respiratory gating system (Real-time position management™ system, Varian, Palo Alto, CA, USA) to evaluate the extent of tumor motion during entire respiratory cycles. The ‘gross tumor volume (GTV)’ was calculated as the sum of the tumor position within a gating phase. It was further expanded by a 5-mm margin and corresponded to the ‘planning target volume (PTV)’ (Fig. 1).

SBRT was planned using five to nine coplanar or non-coplanar beams with energies of 6 to 15 MV (Eclipse™, Varian, Palo Alto, CA, USA). A dose of 10–20 Gy (median, 15 Gy) per fraction was given over 3–4 consecutive days to a total dose of 30–60 Gy (median, 45 Gy) to the isodose line covering the PVT (generally 85–90% of the isodose line) (Fig. 1). The pretreatment tumor position was verified on the basis of fiducial markers detected by the on-board imaging system (On-Board Imager®, Varian, Palo Alto, CA, USA) which is part of a linear accelerator (CL21iX, Varian, Palo Alto, CA, USA) and includes a two-dimensional (2D) kilovoltage radiograph and cone-beam CT.

### CT Imaging

CT scans were obtained with 4 to 64 multidetector CT scanners (Definition AS or Sensation 16 Siemens, Erlangen, Germany; LightSpeed, GE medical Systems, Milwaukee, WI, USAs) in the unenhanced, arterial, portal, and delayed venous contrast-enhanced phases (Table 1). The effective tube current time product was generally 200 mAs. The other parameters were described in detail in Table 1. Patients were given 2 mL/kg of iopromide (Ultravist 370; Schering, Berlin, Germany) or iopamidol (Isovue 370; Bracco, Milan, Italy) intravenously at a rate of 4 mL/sec via the antecubital vein. Arterial phase images were obtained using a bolus tracking technique with a trigger enhancement threshold at the upper abdominal aorta of 100 HU. After the threshold was reached, a diagnostic delay time of 25 seconds was used for the arterial phase. Portal and delayed phase images were obtained 72 and 180 seconds, respectively, following contrast injection.

**Table 1:** CT scanning parameters.

Parameters	Sensation 16 (n = 60)	LightSpeed VCT (n = 52)	LightSpeed plus (n = 40)	LightSpeed 16 (n = 16)	LightSpeed QX/I (n = 12)	Definition AS+ (n = 3)
Number of detectors	16	64	4	16	16	4
Effective tube current-time production (mAs)†	200	200	200	200	250	200
Tube voltage (kVp)	120	120	120	120	120	120
Collimation (mm)	1.5	0.625	1.25	0.625	1.25	1.25
Rotation time (sec)	0.5	0.5	0.6	0.5	0.6	0.6
Pitch	1	1	0.75	1	0.75	0.75

† Automatic tube current modulation was used.

### Interpretation of CT Images

**Figure 2:**
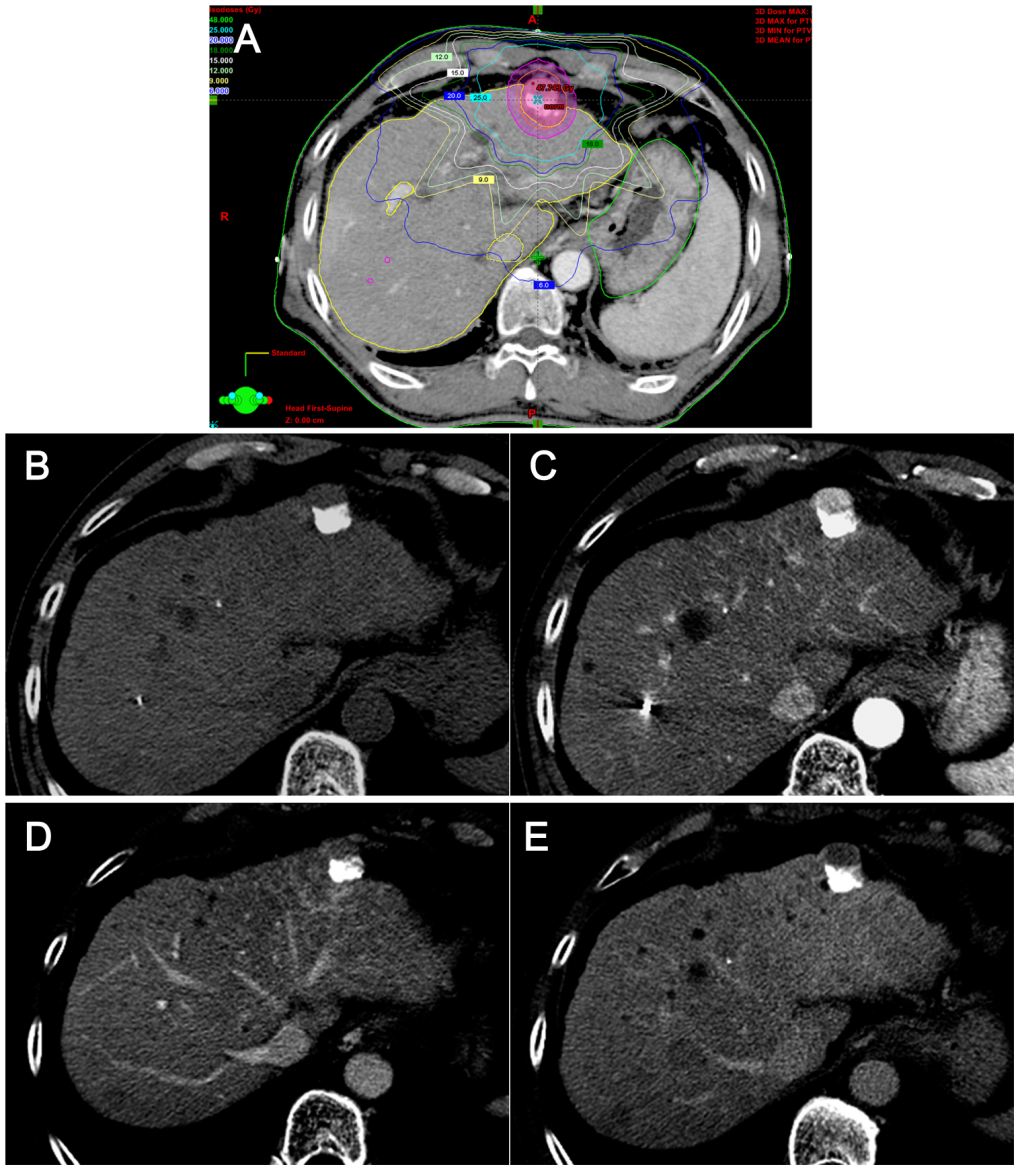
Typical hepatic parenchymal change seen on one month after SBRT. (A) As seen on the isodose curve for planning SBRT with a total dose of 45 Gy, the planning target volume (PTV, purple circle) is expanded to cover the gross tumor volume (red circle). The irradiated hepatic parenchyma is delineated corresponding to the region inside the 15-Gy isodose line, but outside of the PTV boundary. (B-E) The target tumor (arrowheads) in the left lateral segment of the liver, was treated with transarterial chemoembolization prior to SBRT. Residual tumor is noted (arrowheads) with arterial enhancement and washout on the portal and delayed phases adjacent to lipiodol. One-month f/u CT images after SBRT demonstrate that there is no significant change in the irradiated hepatic parenchyma or in the tumor (arrowheads) in any phase including noncontrast (B), arterial (C), portal (D), and delayed (E) phases. A fiducial marker (arrows) is also seen.

The images were evaluated in consensus by two abdominal radiologists (M.J.P and S.Y.K with 5 and 9 years, respectively, of clinical experience in abdomen CT). Irradiated versus non-irradiated liver parenchyma was delineated in each patient by cross-correlation with the dose-volume histogram of CT for the SBRT treatment plan (Fig. 2). As previous studies [5], [22] suggested a threshold dose for hepatic attenuation differences in irradiated hepatic parenchyma from 13.7 Gy, a 15-Gy isodose curve on the dose-volume histogram was used as the outer boundary for the delineation of irradiated hepatic parenchyma. The calculated dose was verified on the follow-up CTs by visual comparison of the initial isodose lines and the margin of radiation reaction. The 15-Gy boundary also enclosed the tumor volume (PTV) irradiated on the dose-volume histogram. In order to exclude the possible tumor area with sufficient margin, we used PTV on the dose-volume histogram and one-year follow up CTs as a reference. In 55 patients with no recurrent tumor on one-year follow up CT in the irradiated area, we used PTV as a reference line for the possible tumor area. In 6 patients who had residual or recurrent HCCs on one-year follow-up CT in the irradiated area, we used the boundary of residual or recurrent tumors as a reference line for the possible tumor area. Therefore, we defined the irradiated non-tumorous hepatic parenchyma as corresponding to the region inside the 15-Gy isodose line but outside the PTV boundary or outside the boundary of the recurrent tumor on one-year follow up CTs (Fig. 1).

The hepatic attenuation difference (HAD) between the irradiated and non-irradiated hepatic parenchyma was determined on the non-contrast, arterial, portal, and delayed phase images at liver windows (window level, 70–100 HU; window width, 130–150 HU) on each follow-up CT scans [27]. If the presence of HAD was uncertain, the observers measured the CT attenuations of the irradiated and non-irradiated hepatic parenchyma. A difference of at least 10 HU between the two regions was defined as a significant HAD [22], [23].

Temporal changes of HAD within a study patient were evaluated according to the follow-up period. For patients who presented with HAD on the follow-up CTs, the time interval from the treatment to the first appearance of arterial hypervascularity and any form of HAD, was analyzed. For patients showing arterial hypervascularity, the HAD patterns seen on other phases were evaluated in order to identify the imaging features mimicking or differing from HCC.

### Statistical Analyses

The Fisher-Freeman-Halton exact test was used to compare the frequency of HAD according to the follow-up period. In addition, comparisons were made using the same test between the following subgroups: (1) patients treated with SBRT only vs. those treated with TACE in combination with SBRT; (2); patients in Child-Pugh A vs. B and (3) patients with single disease vs. those with multiple lesions. Statistical analysis was performed using software (SPSS, version 19, SPSS, Chicago, IL, US). For all statistical analyses, a *P*-value <.05 was considered to indicate a significant difference.

## Results

**Figure 3:**
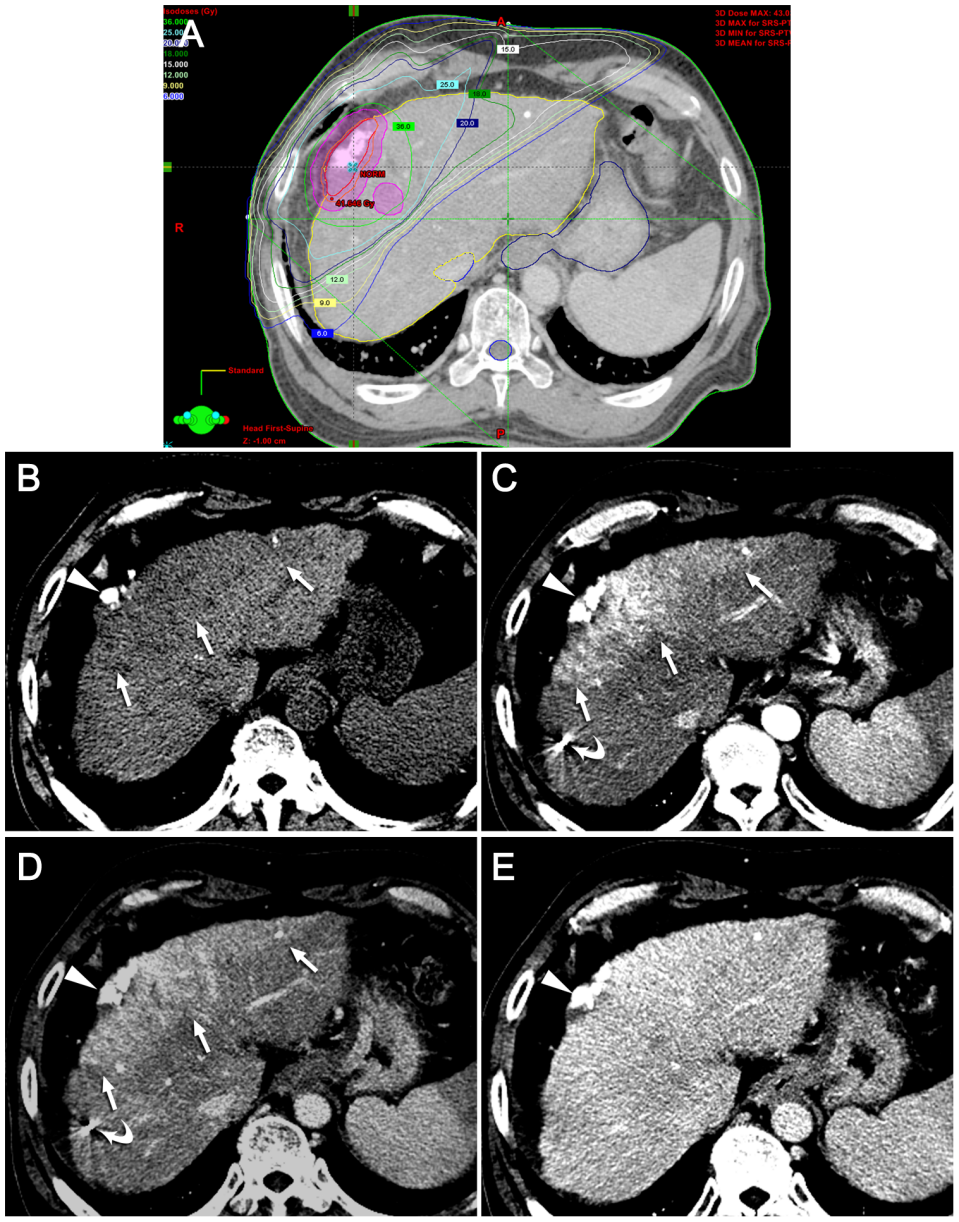
Typical hepatic attenuation difference seen on three months after SBRT. (A) Dose distribution is demonstrated on the isodose curve for SBRT. The planning target volume (PTV, purple circle) as well as the gross tumor volume (red circle) in the peripheral portion of the right lobe of the liver, was treated with 36 Gy of the total radiation dose. The irradiated hepatic parenchyma is delineated corresponding to the region inside the 15-Gy isodose line but outside the PTV boundary. (B-E) Multiphase CT images obtained three months after SBRT depict low attenuation (arrows) on noncontrast CT (B), high attenuation (arrows) on the arterial (C) and portal (D) phases, and isoattenuation on the delayed (E) phase, respectively. The target tumor (arrowheads) in the peripheral portion of the right lobe of the liver was treated using transarterial chemoembolization prior to SBRT. Fiducial markers are also seen (curved arrows).

**Figure 4:**
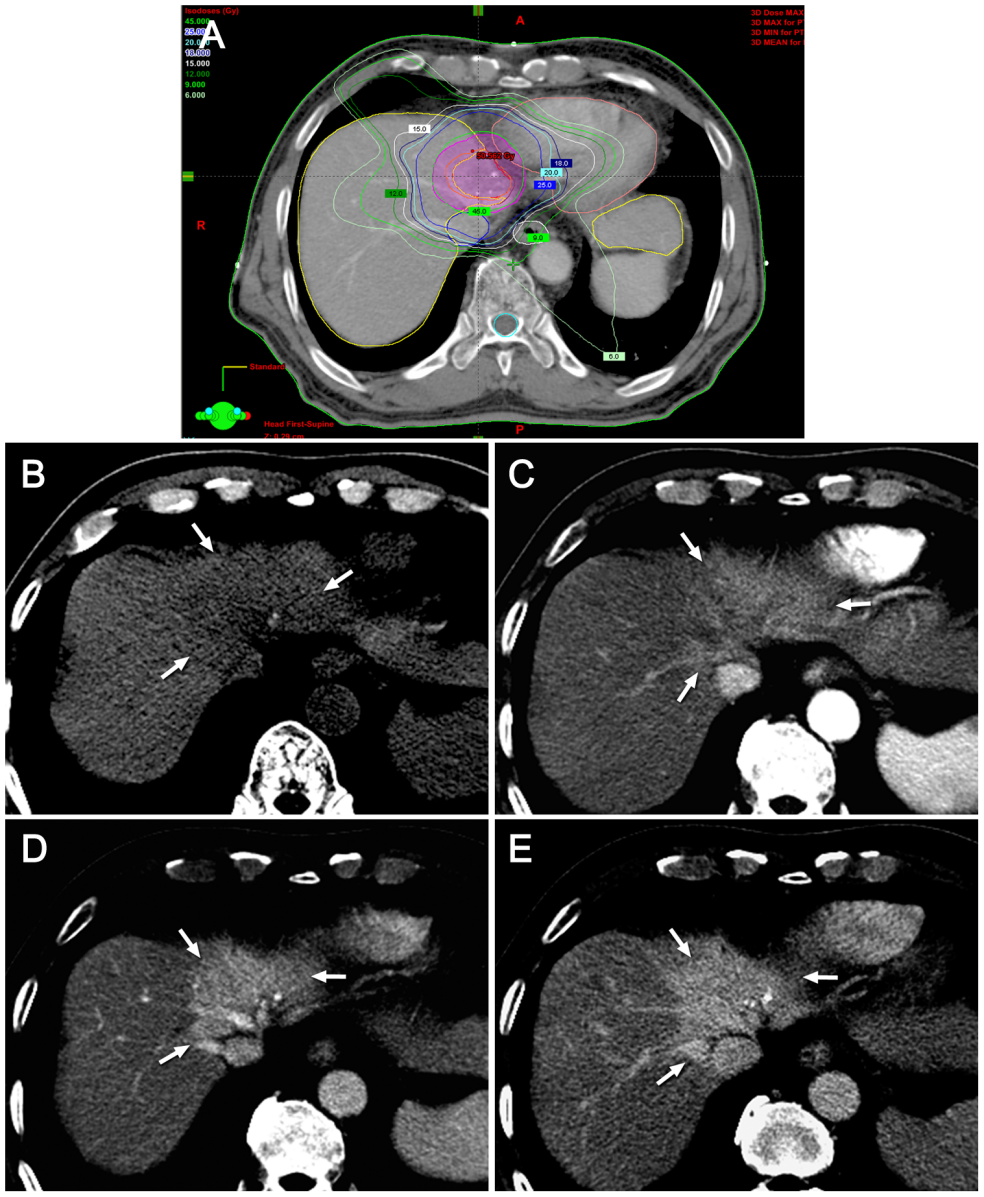
Typical hepatic attenuation difference seen on six months after SBRT. (A) The isodose curve for SBRT depicts the planning target volume (PTV, purple circle) as well as the gross tumor volume (red circle) for the treatment of hepatocelluar carcinoma in left lobe of the liver. The total radiation dose for this patient was 45 Gy. The irradiated hepatic parenchyma is delineated corresponding to the region inside the 15-Gy isodose line but outside the PTV boundary. (B-E) Irradiated hepatic parenchyma appears as low attenuation (arrows) on noncontrast CT (B) and as high attenuation on the arterial (C), portal (D), and delayed (E) phase, respectively.

The CT findings of the irradiated hepatic parenchyma are summarized in Table 2. The areas and shapes of HAD corresponded well to the 15-Gy isodose line seen on the dose-volume histogram of the SBRT treatment plan. Hypervascularity on the arterial phase in the irradiated hepatic parenchyma was observed only in 11.5% (7/61) of our patients on the one-month follow-up CT examination. On subsequent follow-up CT scans, the incidence of arterial hypervascularity increased up to 54.1% (33/61) on the six-month follow-up CT. The temporal changes in arterial hypervascularity were, therefore, statistically significant (*P*<.01). On non-contrast CT, the irradiated hepatic parenchyma increasingly showed low attenuation compared to the nonirradiated hepatic parenchyma, which was seen in 26.2% (16/61) on the one-month follow-up CT and in 78.7% (48/61) on six-month follow-up CT. On the portal phase, the irradiated hepatic parenchyma was commonly seen as hyperattenuation after the three-month follow-up period; 47.5% (29/61) in three months and 60.7% (37/61) in six months after SBRT. Delayed phase images demonstrated isoattenuation or hyperattenuation in the majority of cases during any follow-up period. Hypoattenuation on the delayed phase was rarely seen on the follow-up CT scanning, i.e. 1.6% (1/61) on one month, 3.3% (2/61) on three-month, and none on the six-month follow-up CT scans. The temporal changes in the enhancement patterns on the non-contrast, portal, and delayed phases were also statistically significant. The typical image features on each follow-up period were presented in Fig. 2–4.

**Table 2:** Multiphase CT attenuation in the irradiated hepatic parenchyma in 61 patients.

Time interval	CT attenuation	Phase			
		Non-contrast	Arterial	Portal	Delayed
**1 month**	Low	16 (26.2%)	3 (4.9%)	9 (14.8%)	1 (1.6%)
	Iso	45 (73.8%)	51 (83.6%)	44 (72.1%)	52 (85.2%)
	High	0 (0%)	7 (11.5%)	13.1 (8%)	8 (13.1%)
**3 months**	Low	41 (67.2%)	7 (11.5%)	12 (19.7%)	2 (3.3%)
	Iso	20 (32.8%)	26 (42.6%)	20 (32.8%)	29 (47.5%)
	High	0 (0%)	28 (45.9%)	29 (47.5%)	30 (49.2%)
**6 months**	Low	48 (78.7%)	5 (8.2%)	8 (12.3%)	0 (0%)
	Iso	12 (19.7%)	23 (37.7%)	16 (24.7%)	30 (49.2%)
	High	1 (1.6%)	33 (54.1%)	37 (60.7%)	31 (50.8%)
***P*** **Values**		<.01	<.01	<.01	<.01`

Data are number of patients with percentages in parentheses. Sum of the percentages may not be 100% due to rounding of the numbers.

When the temporal changes in the enhancement patterns within a patient were analyzed, the time intervals between SBRT and the first appearance of arterial hypervascularity in the irradiated hepatic parenchyma were one month in 11.5 (7/61)% of the patients, three months in 34.4% (21/61), and six months in 14.8% (9/61). At the time of the first appearance of arterial hypervascularity, the same areas were seen as low attenuation in 57.1% (4/7), 57.1% (12/21), and 11.1% (1/9) of the patients on non-contrast CT. However, irradiated parenchyma was not seen in any of these patients as low attenuation on either the portal or the delayed phase. The time intervals between SBRT and the first appearance of any form of HAD in the irradiated hepatic parenchyma were one month in 39.3% (24/61), three months in 55.7% (34/61), and six months in 3.3% (2/61). In only one of 61 study patients (1.6%) did the irradiated hepatic parenchyma appear completely isoattenuated on any phase of CT during the follow-up period.

In the subgroup analysis, the group treated with SBRT only and the group with combined therapy of TACE and SBRT, were similar in their contrast enhancement patterns in the irradiated hepatic parenchyma: the incidence of arterial hypervascularity increased gradually until six months after SBRT up to 57.1% (24/42) in those with SBRT only, while up to 47.4% (9/19) in those with combination therapy of TACE and SBRT; on the portal and delayed phase images, the majority of the irradiated hepatic parenchyma appeared iso- or hyper-attenuation (Table 3). The temporal changes in the enhancement patterns on each phase were statistically significant for both groups (*P*<.01) except for the portal phase of the combined therapy subgroup (*P* = 0.1). In the subgroup of Child-Pugh class A, the incidence of arterial hypervascularity in the irradiated hepatic parenchyma increased significantly during the follow up period from 10% (5/50) to 52.5% (21/40) (*P*<.01) (Table 4). Contrast washout on the delayed phases images was rarely seen in this group (only 1 patient on the 3-month follow-up CT). Similar phase-specific enhancement patterns were observed in the subgroup of Child-Pugh class B. However, temporal changes in the subgroup of Child-Pugh class B were not statistically significant, possibly because of the small number of the patients in each follow-up division (Table 4). Contrast enhancement patterns between the subgroups of a single HCC (n = 50) and multiple HCCs (n = 11) were also similar at each specific follow-up period (Table 5). The temporal changes were statistically significant in the patients with a single HCC, although the temporal changes in the arterial and delayed phase images in the patients with multiple HCCs did not show statistical significance, possibly due to the small number of the patients with multiple HCCs.

**Table 3:** Multiphase CT attenuation in the irradiated hepatic parenchyma in between patients treated with SBRT only vs. those treated with TACE in combination with SBRT.

Subgroups	Time interval	CT attenuation	Phase			
			Noncontrast	Arterial	Portal	Delayed
**Patients**	1 month	Low	10 (23.8%)	2 (4.8%)	5 (11.9%)	0 (0%)
**with**		Iso	32 (76.2%)	35 (83.3%)	33 (78.6%)	38 (90.5%)
**SBRT**		High	0 (0%)	5 (11.9%)	4 (9.5%)	4 (9.5%)
**only**	3 months	Low	31 (73.8%)	2 (4.8%)	6 (14.3%)	1 (2.4%)
**(n = 42)**		Iso	11 (26.2%)	20 (47.6%)	12 (28.6%)	21 (50.0%)
		High	0 (0%)	20 (47.6%)	24 (57.1%)	20 (47.6%)
	6 months	Low	33 (78.6%)	2 (4.8%)	4 (9.5%)	0 (0%)
		Iso	9 (21.4%)	16 (38.1%)	12 (28.6%)	25 (59.5%)
		High	0 (0%)	24 (57.1%)	26 (61.9%)	17 (40.5%)
	*P* Values		<.01	<.01	<.01	<.01
**Patients**	1 month	Low	6 (31.6%)	1 (5.3%)	4 (21.1%)	1 (5.3%)
**with**		Iso	13 (68.4%)	16 (84.2%)	11 (57.9%)	14 (73.7%)
**combined**		High	0 (0%)	2 (10.5%)	4 (21.1%)	4 (21.1%)
**therapy**	3 months	Low	10 (52.6%)	5 (26.3%)	6 (31.6%)	1 (5.3%)
**(n = 19)**		Iso	9 (47.4%)	6 (31.6%)	8 (42.1%)	8 (42.1%)
		High	0 (0%)	8 (42.1%)	5 (26.3%)	10 (52.6%)
	6 months	Low	15 (78.9%)	3 (15.8%)	4 (21.1%)	0 (0%)
		Iso	3 (15.8%)	7 (36.8%)	4 (21.1%)	5 (26.3%)
		High	1 (5.3%)	9 (47.4%)	11 (57.9%)	14 (73.7%)
	*P* Values		<.01	<.01	0.1	<.01

Data are number of patients with percentages in parentheses. Sum of the percentages may not be 100% due to rounding of the numbers.

**Table 4:** Multiphase CT attenuation in the irradiated hepatic parenchyma in subgroups according to Child-Pugh class A vs. B.

Subgroups	Time interval	CT attenuation	Phase			
			Noncontrast	Arterial	Portal	Delayed
**Child-Pugh**	1 month	Low	13 (26.0%)	3 (6.0%)	8 (16.0%)	1 (2.0%)
**Class A**	(n = 50)	Iso	37 (74.0%)	42 (84.0%)	36 (72.0%)	43 (86.0%)
		High	0 (0%)	5 (10.0%)	6 (12.0%)	6 (12.0%)
	3 months	Low	32 (69.6%)	6 (13.0%)	11 (23.9%)	2 (4.3%)
	(n = 46)*	Iso	14 (30.4%)	19 (41.3%)	15 (32.6%)	20 (43.5%)
		High	0 (0%)	21 (45.7%)	20 (43.5%)	24 (52.2%)
	6 months	Low	32 (80.0%)	5 (12.5%)	6 (15.0%)	0 (0%)
	(n = 40)†	Iso	8 (20.0%)	14 (35.0%)	10 (25.0%)	16 (40.0%)
		High	0 (0%)	21 (52.5%)	24 (60.0%)	24 (60.0%)
	*P* Values		<.01	<.01	<.01	<.01
**Child-Pugh**	1 month	Low	3 (27.3%)	0 (0%)	1 (9.1%)	0 (0%)
**Class B**	(n = 11)	Iso	8 (72.7%)	9 (81.8%)	8 (72.7%)	9 (81.8%)
		High	0 (0%)	2 (18.2%)	2 (18.2%)	2 (18.2%)
	3 months	Low	8 (57.1%)	1 (7.1%)	1 (7.1%)	0 (0%)
	(n = 14)*	Iso	6 (42.9%)	7 (50.0%)	5 (35.7%)	8 (57.1%)
		High	0 (0%)	6 (42.9%)	8 (57.1%)	6 (42.9%)
	6 months	Low	13 (72.2%)	0 (0%)	2 (11.1%)	0 (0%)
	(n = 18)†	Iso	4 (22.2%)	9 (50.0%)	6 (33.3%)	12 (66.7%)
		High	1 (5.6%)	9 (50.0%)	10 (55.6%)	6 (33.3%)
	*P* Values		.05	.22	.21	.47

Data are number of patients with percentages in parentheses. Sum of the percentages may not be 100% due to rounding of the numbers.

* One patient with Child-Pugh class C was excluded on 3 months follow up.

† Three patients with Child-Pugh class C was excluded on 6 months follow up.

**Table 5:** Multiphase CT attenuation in the irradiated hepatic parenchyma in subgroups according to single HCC vs. multiple HCCs.

Subgroups	Time interval	CT attenuation	Phase			
			Noncontrast	Arterial	Portal	Delayed
**Single**	1 month	Low	14 (28.0%)	3 (6.0%)	8 (16.0%)	1 (2.0%)
**(n = 50)**		Iso	36 (72.0%)	42 (84.0%)	36 (72.0%)	42 (84.0%)
		High	0 (0%)	5 (10.0%)	6 (12.0%)	7 (14.0%)
	3 months	Low	32 (64.0%)	7 (14.0%)	12 (24.0%)	2 (4.0%)
		Iso	18 (36.0%)	21 (42.0%)	16 (32.0%)	23 (46.0%)
		High	0 (0%)	22 (44.0%)	22 (44.0%)	25 (50.0%)
	6 months	Low	39 (78.0%)	5 (10.0%)	8 (16.0%)	0 (0%)
		Iso	10 (20.0%)	18 (36.0%)	13 (26.0%)	23 (46.0%)
		High	1 (2.0%)	27 (54.0%)	29 (58.0%)	27 (54.0%)
	*P* Values		<.01.	<.01	<.01	<.01
**Multiple**	1 month	Low	2 (18.2%)	0 (0%)	1 (9.1%)	0 (0%)
**(n = 11)**		Iso	9 (81.8%)	9 (81.8%)	8 (72.7%)	10 (90.9%)
		High	0 (0%)	2 (18.2%)	2 (18.2%)	1 (9.1%)
	3 months	Low	9 (81.8%)	0 (0%)	0 (0%)	0 (0%)
		Iso	2 (18.2%)	5 (45.5%)	4 (36.4%)	6 (54.5%)
		High	0 (0%)	6 (54.5%)	7 (63.6%)	5 (45.5%)
	6 months	Low	9 (81.8%)	0 (0%)	0 (0%)	0 (0%)
		Iso	2 (18.2%)	5 (45.5%)	3 (27.3%)	7 (63.6%)
		High	0 (0%)	6 (54.5%)	8 (72.7%)	4 (36.4%)
	*P* Values		<.01	.17	.04	.24

Data are number of patients with percentages in parentheses. Sum of the percentages may not be 100% due to rounding of the numbers.

## Discussion

Our study demonstrated that HAD in the irradiated non-tumorous hepatic parenchyma occurred in almost all of our patients after SBRT during the six-month follow-up period. Arterial hypervascularity in the irradiated non-tumorous hepatic parenchyma was observed in the considerable population of the patients and the incidence increased during the follow-up period, i.e. 11.5% of patients in one month, 45.9% in three months, and 54.1% in six months. According to the modified RECIST criteria recently suggested as a reliable method for assessing the tumor response in HCC clinical trials by American Association for the Study of Liver Diseases (AASLD)-Journal of the National Cancer Institute (JNCI), changes in the area of arterial enhancement is considered important in the evaluation of tumor response.[25] Therefore, arterial hypervascularity in the irradiated non-tumorous hepatic parenchyma is clinically important as it can create difficulties in evaluating the tumor response of HCC. In addition, the temporal changes of arterial hypervascularity in the irradiated hepatic parenchyma could also interfere with the accurate evaluation of treatment response. In our study, the first appearance of arterial hypervascularity in the irradiated hepatic parenchyma reached its peak on the three-month follow up CTs. As the treatment response of HCC after SBRT also appeared after three months [5], [22], [23], the temporal changes of the treatment response seen in a tumor could coincide with that of the arterial hypervascularity seen in the irradiated hepatic parenchyma.

In this context, post-SBRT arterial hypervascularity of non-tumorous hepatic parenchyma could be misinterpreted as pseudoprogression, a term originally applied to high-grade glioma showing a temporal increase in contrast-enhancing lesion size after concurrent radiation therapy and temozolomide [28]. However, on multiphase contrast-enhanced CT, we found that some features of the contrast-enhancement patterns of the non-tumorous hepatic parenchyma related to SBRT, differed from the known typical findings of HCC [18]-[21], even though we did not perform tumor response evaluation itself and our study was limited in the evaluation of tumor recurrence due to lack of pathologic proof. Based on our observation in this study, and the lack of contrast wash-out seen on the delayed phase would be suggested as the helpful features for differentiating radiation-induced changes in the non-tumorous hepatic parenchyma and tumor progression, when we encounter arterial hypervascularity in the irradiated liver after SBRT. These findings are consistent with those of previous studies reporting imaging findings after SBRT [22], [23].

The changes we found in the contrast enhancement patterns in the irradiated hepatic parenchyma after SBRT differed from those seen after conventional radiation therapy [9]–[14]. In particular, hepatic arterial hypervascularity has not yet been reported following conventional radiation therapy [9]–[14]. This difference could possibly be explained by the differences in radiation dose and distribution in SBRT and conventional radiation therapy. Nevertheless, the underlying histolopathologic mechanism of SBRT-related change in the irradiated non-tumorous hepatic parenchyma has not yet been clearly elucidated. To our knowledge, there has been no published report indicating the difference in the underlying histolopathologic mechanism between SBRT and conventional radiation therapy, although venoocclusive disease has been suggested as the mechanism of the parenchymal changes seen after SBRT [10], [29]. However, the study regarding the pathologic change in the liver after SBRT analyzed only two specimens [29] as it was difficult to obtain surgical specimens after SBRT. To date, SBRT is generally recommended as a treatment option for patients with inoperable HCC [3]–[5], [7]. However, a recent study recommends SBRT as a bridge to transplantation in transplantation-eligible patients [5]. When this application becomes increasingly used, further studies with extensive histopathologic correlation can be performed in order to determine the difference in the underlying mechanism which explains the different imaging findings obtained after SBRT and conventional radiation therapy.

As some patients of our study population were treated with combination of TACE and SBRT, we investigated and compared the effects of the combined therapy versus SBRT only on the contrast enhancement patterns and their temporal changes by performing the subgroup analysis. These two subgroups were similar in their contrast enhancement patterns and temporal changes. Two additional subgroup analyses (single vs. multinodular lesions and Child-Pugh A vs. B) also revealed contrast enhancement patterns similar between the paired subgroups across the follow-up periods.

Our study has several limitations. First, lack of pathologic proof for the areas of presumed irradiated nontumorous hepatic parenchyma in all patients. Considering that the current indication of SBRT is mainly confined to the patients with HCC as mentioned in the previous paragraph, it was difficult to obtain surgical specimens after SBRT [3]–[5], [7]. Thus, we tried to exclude the possible tumor area using both planning CT for SBRT and one-year follow-up CTs. However, lack of pathologic correlation would decrease the reliability of our study regarding the differentiating post-radiotherapy arterial hypervascularity and residual tumor. Second, to delineate the irradiated hepatic parenchyma, we correlated the isodose line of the SBRT treatment planning CT with the follow-up CT images using visual comparison. For the exact and objective definition of the irradiation of hepatic parenchyma, a co-registration technique over multiple time points would be required.

In conclusion, after SBRT, arterial hypervascularity of non-tumorous hepatic parenchyma was increasingly seen on follow-up CT scans and it could potentially interfere with an accurate treatment response. The lack of wash-out on the delayed phase and the shape and area of the radiation field would, therefore, help to differentiate radiation-induced arterial hypervascularity from residual HCC.
